# Attempted ablation of an orbital cyst in a dog with 1% polidocanol using fluoroscopy and contrast cystography

**DOI:** 10.1111/vop.13313

**Published:** 2025-01-07

**Authors:** Marissa R. Matthews, Katrina E. V. Jones, Hannah Flanders, Kathryn Wotman, Allison Cleymaet, Brian A. Scansen

**Affiliations:** ^1^ Department of Clinical Sciences, College of Veterinary Medicine and Biomedical Sciences Colorado State University Fort Collins Colorado USA; ^2^ Comparative Ophthalmology, Department of Clinical Sciences, College of Veterinary Medicine and Biomedical Sciences Colorado State University Fort Collins Colorado USA; ^3^ Cardiology & Cardiac Surgery, Department of Clinical Sciences, College of Veterinary Medicine and Biomedical Sciences Colorado State University Fort Collins Colorado USA

**Keywords:** canine, chemical ablation, conjunctival cyst, foam, ocular, post‐enucleation

## Abstract

A 4‐year‐old female spayed mixed‐breed dog received enucleation surgery of the right eye in 2018 following the diagnosis of glaucoma. The patient was presented in 2021 for recurrent swelling of the right orbit. Ultrasound confirmed the presence of a cystic structure, and chemical ablation with 1% polidocanol (compounded, Stokes Pharmacy, Mt. Laurel, NJ) and contrast cystography was elected for treatment. Iodinated contrast was injected under fluoroscopy and after no leakage was noted, the contrast was aspirated from the orbital cyst and immediately followed by an injection of 1% polidocanol solution. The orbital cyst collapsed within 24 h but returned 25 days post‐injection. A second attempt was made for chemical cyst wall ablation with 1% polidocanol foam and ultrasound guidance. The orbital cyst returned 12 weeks post 1% polidocanol foam injection, and the patient presented for surgical removal. Histopathology revealed an orbital conjunctival inclusion cyst associated with granulomatous and lymphoplasmacytic inflammation. Histopathology confirmed that the draining cyst was lined by conjunctiva and likely represented conjunctival remnants from the previous enucleation. This report describes fluoroscopic guidance for sclerotherapy in the treatment of a post‐enucleation orbital cyst in a dog. Contrast cystography may be useful to verify catheter position and demonstrate cyst margins prior to injection. This case report highlights the importance of complete conjunctival excision during enucleation.

## 1 INTRODUCTION

Enucleation is a common surgical procedure performed as a palliative treatment for blind and painful eyes not amenable to other treatments. The most common immediate postoperative complication of the procedure is hemorrhage and swelling.^1^ Formation of an orbital cyst is an uncommon long‐term complication of enucleation and results from retention of orbital secretory tissues (nictitans gland and conjunctival tissue).^2^ Incomplete removal of secretory and adnexal tissues is more common with the subconjunctival enucleation technique.^3^ Additionally, ocular trauma has been implicated as a predisposing factor for postoperative orbital cysts.^2^ Retention of cystic structures can be unaesthetic and may lead to the formation of draining tracts.^2^, ^3^ The gold standard treatment of an orbital cyst is surgical excision of the cyst and any remaining secretory tissues^4^ which requires general anesthesia. Additional surgical techniques that have been reported include excision after visualization of the cyst with dye, needling with a syringe, using a Nd:YAG laser or argon laser, thermal or chemical cautery, high‐frequency radio‐wave electrosurgery, cryotherapies, and atmospheric low‐temperature plasma.^5^, ^6^

One percent polidocanol (hydroxypolyathoxydodecan), a synthetic long‐chain fatty alcohol, is a commonly used sclerosing agent; it induces lysis of the cyst lining, leading to fibrosis.^7^ Successful sclerotherapy with 1% polidocanol has been reported for orbital sialoceles, vascular malformations, dacryops, and lymphangiomas in humans, horses, and dogs.^7^, ^8^, ^9^, ^10^ The additional analgesic effect results in a relatively painless injection.^11^ It is a frequent practice in human medicine to combine the sclerosing agent with contrast to visualize and prevent inadvertent sclerosis of nontarget tissues.^12^, ^13^

This case report describes a 4‐year‐old female spayed mixed‐breed dog that presented with an orbital cyst post‐enucleation; two attempts were made to obliterate the cyst using a percutaneous injection of 1% polidocanol. A literature search failed to identify any prior reports of the use of the sclerosing agent 1% polidocanol under contrast cystography for the attempted treatment of a post‐enucleation orbital cyst in a dog.

## 2 CASE SUMMARY

A 4‐year‐old female spayed mixed‐breed dog was presented to the ophthalmology service at Colorado State University's Veterinary Teaching Hospital (CSU‐VTH) in December 2021 for a recurrent right (OD) orbital swelling. Historically, the patient received enucleation surgery OD in 2018 following diagnosis of glaucoma. An orbital swelling developed in February 2019, and clear‐yellow fluid was aspirated by the primary veterinarian. The orbit required manual drainage via aspiration every 6 to 9 months, until 6 months prior to presentation, where the orbit required aspiration every 3 to 4 weeks.

**FIGURE 1:**
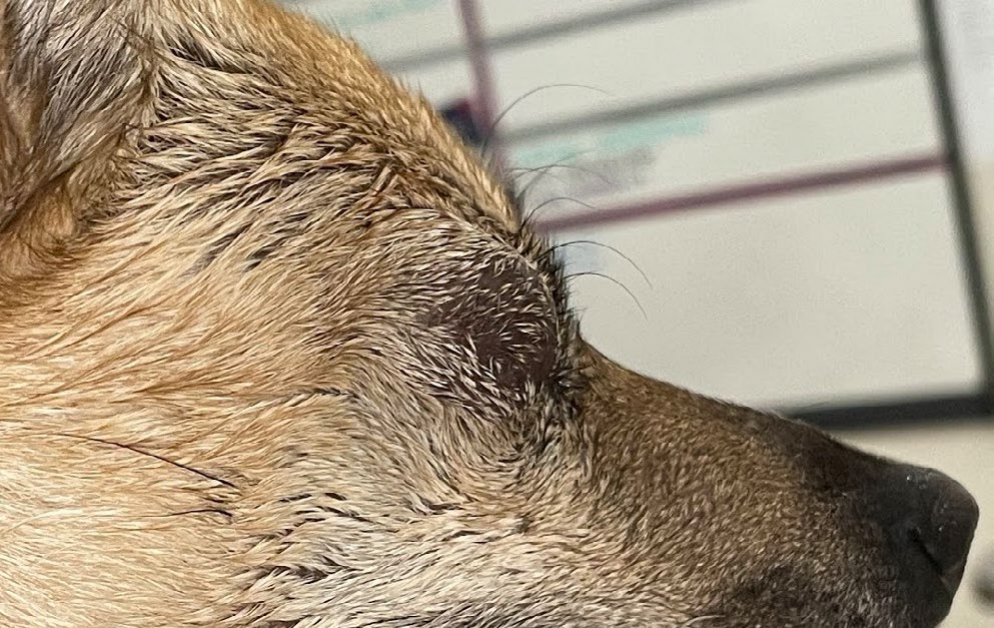
Gross photo of the 4‐year‐old female spayed mixed‐breed dog that presented with a suspected orbital cyst post‐enucleation in the location of the right eye (OD).

**FIGURE 2:**
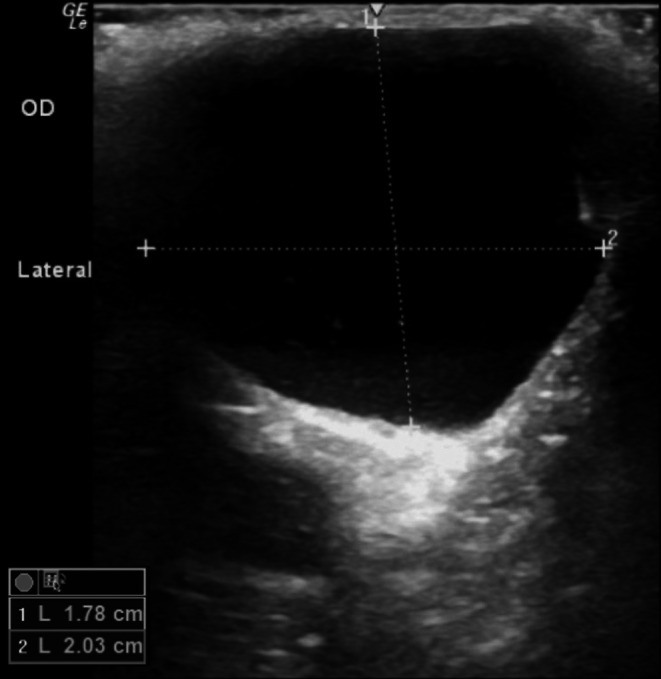
Ultrasonography image of a well‐demarcated anechoic structure with moderate amounts of echogenic debris at the site of the previous enucleation, confirming an orbital cyst.

Ophthalmic examination revealed moderate flocculent, soft, and nonpainful swelling of the orbit OD (Figure 1). The left eye (OS) was normal apart from the presence of multifocal axial punctate refractile white corneal opacities, consistent with metabolic deposits. Ultrasound OD confirmed the presence of a cystic structure measuring 2.03 cm by 1.78 cm with moderate echogenic debris (Figure 2). The patient was given an ophthalmic diagnosis of orbital cyst OD. A recommendation was made for either orbital exploration and surgical excision or contrast cystography with chemical ablation. The owner elected the latter, and written consent was obtained for treatment and for publication of the dog's picture.

**FIGURE 3:**
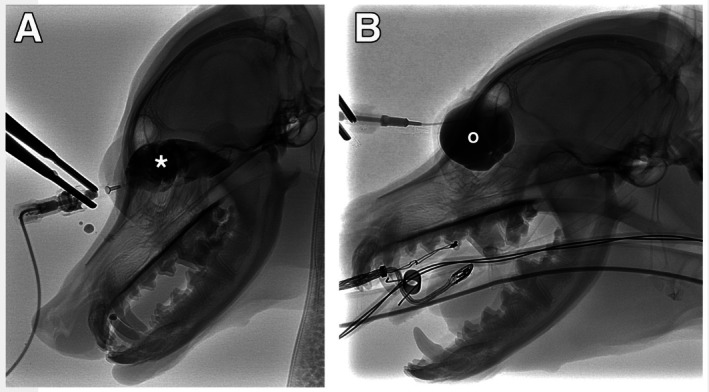
Fluoroscopic images showing iodinated contrast (iohexol 240 mg/mL) in the region of the orbital cyst. (A) Cystography during the first attempt demonstrated that the 22‐G catheter was not in the cyst, as contrast (*) extravasated after the patient moved, and 1% polidocanol was therefore not injected. (B) 6 weeks later, repeat cystography demonstrated the location and extent of the orbital cyst (o), without extravasation or drainage, and 1% polidocanol was therefore injected into this structure.

A complete blood cell count and serum chemistry profile were unremarkable. Sedation was achieved with butorphanol 0.3 mg/kg IV, acepromazine 10 μg/kg IV, and dexmedetomidine 7.5 μg/kg IV. The patient was positioned in left lateral recumbency, and the right orbital region was clipped and sterilely prepared with dilute betadine. A 22‐gauge catheter was placed in the orbital swelling OD, and 2.5 mL of clear fluid was aspirated. After removal, the patient pawed at the face, causing the catheter to dislodge. A new catheter was placed, and injection of iodinated contrast (iohexol 240 mg/mL) through the catheter under digital subtraction angiography confirmed that the catheter was not in the cyst. The contrast was noted to fill the confines of the orbit with no escape (Figure 3A). The 1% polidocanol was unable to be injected, and the dog was reversed with atipamezole IM. Cytology of the collected fluid revealed a low‐nucleated cellularity fluid with scant proteinaceous debris and scant mineralized debris. Aerobic and anaerobic culture of the fluid revealed no growth.

The patient was re‐presented 6 weeks later for a second attempt at orbital cystography and 1% polidocanol injection. The patient was premedicated with methadone 1 mg/kg IM and dexmedetomidine 5.3 μg/kg IM. General anesthesia was induced with propofol 2.6 mg/kg IV and maintained with inhalation isoflurane. Positioning and sterile preparation were similar on this attempt. A 22‐gauge catheter was advanced into the orbital cyst and 1.5 mL of clear fluid was aspirated. Iodinated contrast (3 mL, iohexol 240 mg/mL) was injected through the catheter under fluoroscopic digital subtraction. The contrast was allowed to dwell for 5 min, and as no extravasation or drainage was noted (Figure 3B), 2 mL of contrast was aspirated from the orbital cyst, immediately followed by a 2 mL injection of 1% polidocanol. The catheter was removed, and the access site was held closed with Bishop–Harmen tissue forceps for 2 to 3 min with no drainage from the access site appreciated. Recovery from anesthesia was uneventful and the patient was discharged the same day with a five‐day course of carprofen 2.2 mg/kg per os (PO or by mouth) every 12 h.

**FIGURE 4:**
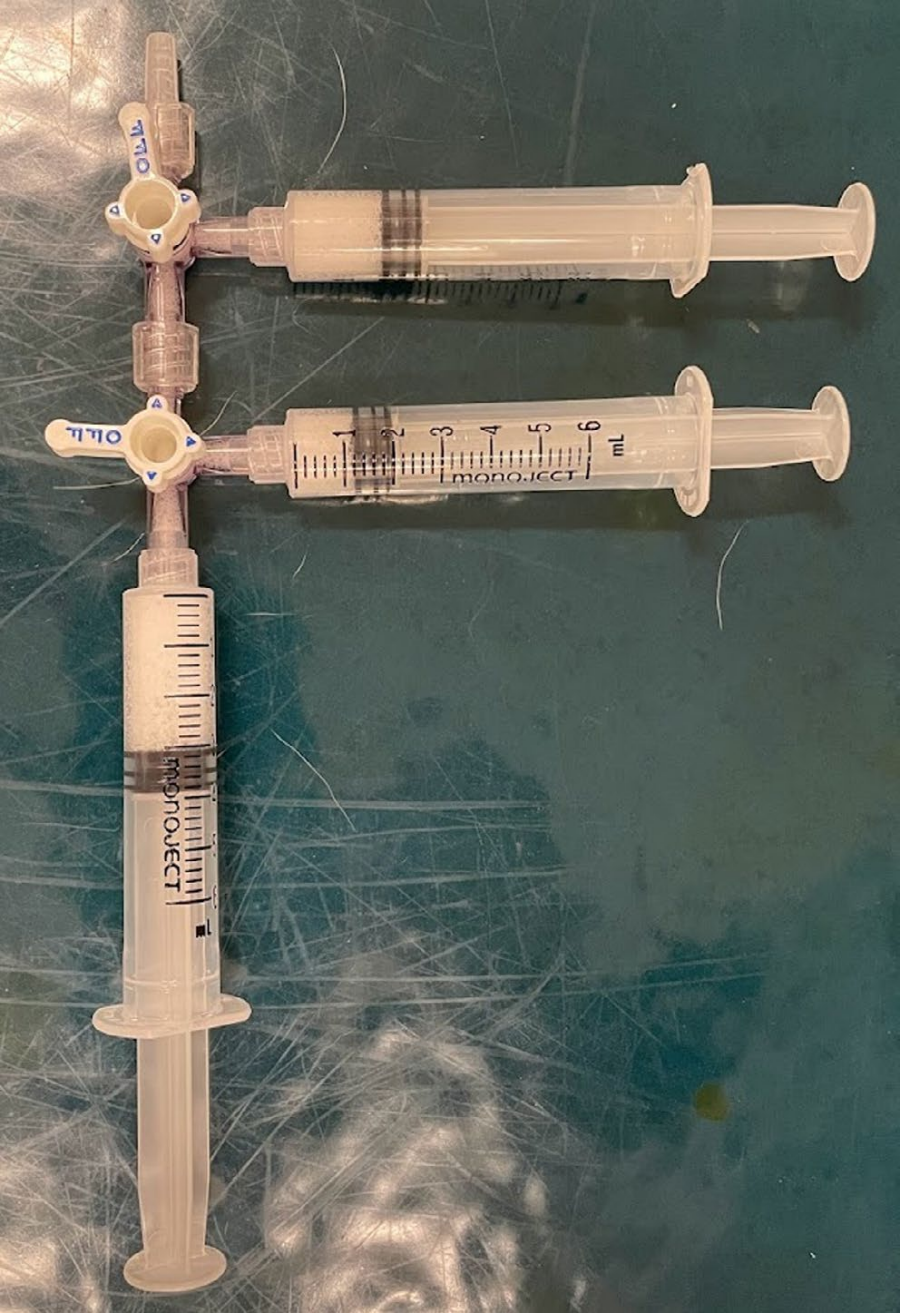
The modified Tessari method for the generation of 1% polidocanol foam.

The orbital cyst collapsed within 24 h but returned 25 days post‐injection. The patient was re‐presented 9 weeks post‐injection for a second orbital cyst ablation with 1% polidocanol. Sedation was induced with methadone 0.3 mg/kg IV and dexmedetomidine 5 μg/kg IV. The skin of the right orbit was aseptically prepared. A local block of 0.5 mL of 2% lidocaine was injected into the subcutaneous tissue in the area of intended catheter placement. A 22‐gauge catheter was advanced into the orbital cyst with ultrasound guidance, and 0.5 mL of clear yellow fluid was aspirated. 1% polidocanol foam was created using the modified Tessari method in a sclerosant–air ratio of 1:4.^14^ Three disposable 6 mL syringes were connected to two medical three‐way stopcocks. One mL of 1% polidocanol was drawn into one syringe, and 4 mL of air were drawn into the other two. The plungers of all the syringes were moved back and forth 20 times to produce sclerosing foam. Four mL of 1% polidocanol foam were injected into the orbital cyst (Figure 4). Atipamezole IM was used for reversal, and the patient recovered uneventfully from sedation. The patient was discharged the same day with a 7‐day course of meloxicam 0.1 mg/kg PO every 24 h.

The orbital cyst was reported to have returned 12 weeks post‐1% polidocanol foam injection. The patient re‐presented 18 weeks post‐injection for surgical removal of the cyst. Premedication was achieved with buprenorphine 0.02 mg/kg IM, Zenalpha (medetomidine and vatinoxan) 6.7 μg/kg IM, and atropine 0.02 mg/kg IM. General anesthesia was induced with propofol 5 mg/kg IV and maintained with inhalation isoflurane. A linear skin incision over the cystic swelling was made with a #15 blade following routine sterile surgical preparation. Tenotomy scissors were used to blunt and sharply dissect the cyst structure. The cyst was inadvertently perforated, and clear fluid was expressed during the procedure. All orbital tissue was removed from the right orbit, and a compartment was not readily identified. The subcutaneous margins of the lid were closed with 4–0 Biosyn monofilament absorbable suture in a subcuticular pattern. An intradermal pattern was used to close the skin with 4–0 Biosyn. The patient recovered uneventfully from anesthesia and was discharged the same day with a 5‐day course of meloxicam 0.1 mg/kg per os (or by mouth) every 24 h and gabapentin 12.3 mg/kg per os (or by mouth) every 8–12 h as needed for pain.

Histopathologic interpretation by the Comparative Ocular Pathology Laboratory of Wisconsin proved the cyst to be composed of multifocally pigmented conjunctival epithelium. Mild granulomatous and lymphoplasmacytic inflammation multifocally subtended the epithelium of the cyst. No neoplastic cells were identified.

## 3 DISCUSSION

Histopathology confirmed the presence of remnant conjunctival tissue creating a cystic structure in this case. Conjunctival tissue contains goblet cells, which produce mucin. The fluid in this structure is presumed to be mucin based on the remnant tissue identified. Differential diagnoses for canine orbital swellings post‐enucleation include cysts, abscesses, seromas, air, and neoplasms. Fluid analysis of the material aspirated from the cyst was consistent with cystic fluid, with no cells present to suggest infectious or neoplastic processes.

1% polidocanol consists of 95% hydroxypolyethoxydodecane, which induces rapid over‐hydration and secondary destruction of endothelial cells.^7^ The remaining 5%, ethyl alcohol, is added as a preservative.^7^ Use in animals has been reported to address spontaneous pneumothorax and hydrothorax by having a sclerosant effect on the mesothelium of porcine pleura via pleurodesis. 1% polidocanol can be used to achieve fibrosis and thrombosis of superficial vessels and cystic structures.^7^ In humans, two or three subsequent percutaneous injections may be necessary to achieve resolution of cystic structures.^15^ One percent polidocanol has been used as both a foam and liquid. Foam is more effective but has a higher incidence of minor secondary side effects, such as pain, inflammation, and skin pigmentation.^16^

One percent polidocanol has been successfully reported to treat a salivary gland mucocele in a dog,^7^ a superficial orbital venous malformation in a horse,^10^ and a suspected nasolacrimal duct cyst in a dog.^17^ The use of repeated injections of 1% polidocanol has been reported to be unsuccessful in the treatment of a presumed dysontogenic orbital cyst in a dog.^18^

The 1% polidocanol injections likely did not have a lasting outcome in this dog because 1% polidocanol targets endothelium, and the tissue lining the cyst was conjunctival epithelium. Another possible reason is that more than two injections may be needed for a lasting outcome.^15^ Total volume injected may have been insufficient as well, though 1.5 mL of 1% polidocanol for an approximately 5 mL volume of cyst was successful in sclerosing a nasolacrimal cyst in a dog.^17^

Contrast cystography was used to confirm catheter placement in the cyst and to confirm no extravasation of the sclerosing agent. This ruled out a draining tract to the cyst and confirmed that the sclerosing agent would not damage local tissue. Contrast cystography may be useful to verify catheter position and demonstrate cyst margins prior to injection of a caustic agent. During the first attempt, where the catheter became dislodged, there was no direct venous drainage beyond the orbit when contrast was placed into the orbital space and not the cyst. On the second attempt, it may have been interesting to repeat fluoroscopy after injecting the sclerosing agent to confirm that it did not leak out, as 1 mL of contrast remained in the cyst.

This case report describes the use of fluoroscopically guided sclerotherapy treatment for a post‐enucleation orbital cyst. The cyst failed to respond to 1% polidocanol therapy, both as a solution and as a foam. Ultimately, the cyst was surgically excised. Therapy likely failed due to 1% polidocanol targeting endothelium while the cyst was lined with epithelium, or more than two injections were needed. Contrast cystography was successful in demarcating cyst margins prior to injection of a caustic agent. This article highlights the importance of complete conjunctival excision during enucleation.

## AUTHOR CONTRIBUTIONS

**Marissa R. Matthews:** Writing – original draft; writing – review and editing. **Katrina E. V. Jones:** Writing – original draft; writing – review and editing. **Hannah Flanders:** Writing – review and editing. **Kathryn Wotman:** Writing – review and editing. **Allison Cleymaet:** Writing – review and editing. **Brian A. Scansen:** Writing – review and editing.

## CONFLICT OF INTEREST STATEMENT

The authors declare no conflicts of interest.

## Data Availability

Data sharing is not applicable to this article as no new data were created or analyzed in this study.
